# Can Chemical Analysis Predict Wine Aging Capacity?

**DOI:** 10.3390/foods10030654

**Published:** 2021-03-19

**Authors:** Andrew L. Waterhouse, Yingxin Miao

**Affiliations:** Viticulture and Enology, University of California, Davis, CA 95616, USA; yxmiao@ucdavis.edu

**Keywords:** oxidation, premox, shelf life, assay

## Abstract

Oxidation is the limiting factor in wine aging, and recently some famous wines have exhibited unexpected premature oxidation. Antioxidant assays may provide a means to assess a wine’s aging potential by measuring its capacity to chemically reduce reagent components. Correlations between antioxidant activity and wine components have the highest value with flavanols, notable for their catechol and phloroglucinol moieties. Both FRAP and DPPH based methods respond strongly to catechol groups, but these functional groups do not protect wine from oxidation. An ideal assay for wine aging capacity would respond selectively to thiols, phloroglucinol moieties, SO_2_ and other antioxidants capable of reducing quinones. A definitive test will be to compare the various assays against the shelf life of a number of commercial wines.

## 1. Introduction

Aging capacity is an essential factor in quality wine. The aging capacity of white Burgundy from the vintage of 1995 to 2000 suddenly dropped, compromising the reputation of older, elegant white Burgundy. The reason was premature oxidation [1]. There were several hypotheses for the reason for premature oxidation, such as less oxygen exposure in the early stages of winemaking, later harvest times, lower sulfur dioxide addition, and corks treated with oxidants. However, all these hypotheses could not fully explain the problem. In addition, the 2003 red Bordeaux showed tired fruit, flabby structure, and were past their prime after ten years of aging. Suggested reasons are the high ripeness of grapes with higher sugar and lower acidity. Lower acidity accelerates the reaction of iron with oxygen, the rate limiting step in oxygen reacting with wine [2]. With global warming, over-ripeness of grapes could become a global issue, leading to a change of grape composition at harvest. The shelf life of some wines could change dramatically. However, the difficult issue is that premature oxidation of a specific new wine is difficult to discern because it usually takes a decade before the problem becomes evident. Thus, accurate prediction of the aging capacity of wine would be very useful.

Wine aging capacity is related to the extent of oxidation with time, where rapid oxidation leads to what is commonly called premox (premature oxidation) in situations where particular wines are expected to age more slowly. The alteration of wine sensory characters due to wine oxidation starts with the degradation of positive aroma, through the development of negative aroma, to browning.

To further understand the wine aging process, the chemical antioxidant compounds in wine were studied. First, oxygen reacts with phenols and yields quinone and hydrogen peroxide. Then, quinones actively react with nucleophiles such as tannin, SO_2_, amino acid, thiols, and flavan-3-ols [3]. Those nucleophiles are antioxidants with different abilities to react with quinones and aldehydes. This has led to the idea that the shelf life of wine might be related to a wine’s antioxidant capacity [4].

**Figure 1:**
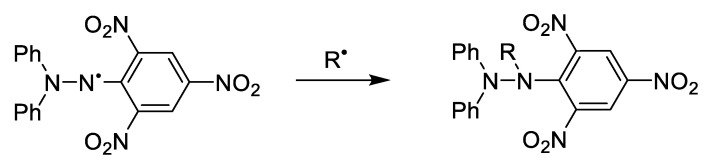
Reaction between DPPH and radical [7].

**Figure 2:**
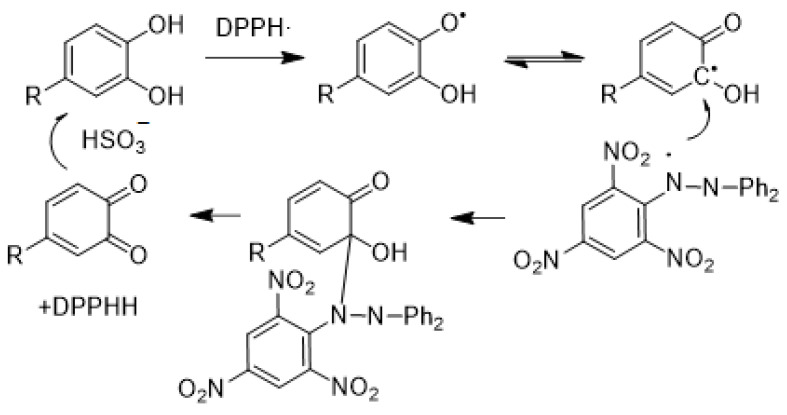
The proposed mechanism for the DPPH• oxidation of catechols [8] DPPH.

The FRAP and DPPH assays are commonly used to determine the antioxidant capacity of samples. In the FRAP assay, an excess of Fe(III), the antioxidants in samples reduce Fe(III) to form Fe(II) which then forms a complex with TPTZ that can be quantified by colorimetric detection at 594 nm. The quantification of Fe(II) indirectly quantifies the antioxidant capacity of samples [5]. In the DPPH assay, DPPH is a free radical with absorbance at 517 nm. It reacts with antioxidants in the sample, quenching the radical and loss of absorbance indicates the amount of antioxidants in the sample [6]. The reaction between DPPH and radical is shown in Figure 1. Take samples that contain both SO2 and antioxidants such as catechols as an example, catechols firstly reduce the DPPH and lead to the loss of absorbancc. Then the radical formed from catechol is trapped by DPPH and then slowly release the quionone. Quionone would be reduced the to catechol by sulfite as shown in Figure 2.

High-performance liquid chromatography (HPLC) assay and Folin-Ciocalteu (FC) assay are used for phenolic compound analysis. HPLC is capable of quantitative and qualitative analysis of a series of single phenol compounds, S- and N- compounds, such as glutathione (GSH) in wine through the process of extraction, separation, ionization of compounds from samples. FC assay uses the reaction between phenols and the reducible compounds in samples, which produces products with absorption at 765 nm [8].

Sensory tests are performed to assess the oxidative state of aged wine. Two types of sensory test have been conducted. One method is to score (0–10) on the honey, farm, hay, woody and floral flavors of wine by tasting panels, key descriptors of the oxidation scale. A second method is to score (0–20) on the similarity between samples and an oxidized wine. Comparison between the results of sensory tests and chemical tests could indicate the correlation between chemical changes and sensory changes. The sensory test could inform whether there is a correlation between chemical tests and the formation of oxidized flavors.

This review will explore the possibility of predicting wine aging capacity with existing methods that measure the antioxidant capacity. The mechanisms of these tests will be described to see how they respond to substances known as preservatives. Comparison between the results of those methods, and some tasting results will be presented.

## 2. Oxidation and Antioxidant Pathways

There are two principal oxidants that arise from the primary oxidation reactions, the quinones and aldehydes. The former arise from the oxidation of catechol-type functional groups, and these are common among the phenolics in wine. The quinones are moderately unstable and if there is nothing available, they will decompose over a number of hours in solution, and so these are not available to purchase, but for experiments can be made just prior to use. Their preparation is very simple-oxidize the catechol with periodate [9].

**Figure 3:**
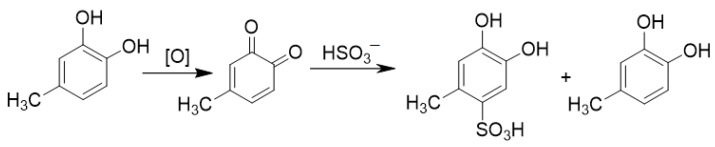
The oxidation of 4-methylcatechol by a generic oxidant, followed by reduction with and addition by sulfur dioxide, reversing the process of oxidation.

In the case of the quinones, antioxidant act by reducing the quinone back to the catechol; some, like ascorbate, do this directly, and some react as nucleophiles which means the catechol is now substituted on one position by the antioxidant. The reaction with sulfite is a good example since both reactions are observed, as shown in Figure 3.

Other wine nucleophiles that participate in this antioxidation pathway include the A-ring of some flavonoids, due to its phloroglucinol configuration of alternative oxygen atoms, and thiols such as glutathione and cysteine. Other phenolic compounds, for instance caffeic acid, which has only a catechol group, is not a particularly good antioxidant. It is not a good nucleophile, and if it reacted with a quinone, would probably exchange oxidation giving rise to a different quinone, not quenching it [10]. Therefore, an ideal antioxidant test would not be sensitive to the reducing capacity of the catechols, but only to the nucleophilic or quinone reducing power of samples.

**Figure 4:**
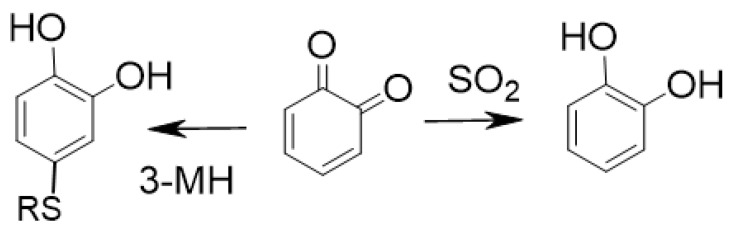
Quinones react quickly with desirable thiols such as 3-MH, but SO_2_ can prevent the loss of 3-MH by reacting instead, acting as a sacrificial antioxidant.

In the absence of antioxidants, the quinones will react with other substances, many being flavor molecules, and compromise some desirable flavors. For instance, an important aroma substance, with a citrusy note, is 3-mercaptohexanol (3-MH). This will react quickly with quinones, but so does SO_2_, and the SO_2_ scavenges the quinone, preserving the fruity aroma of the 3-MH (Figure 4). Thus for white wines, the fruity flavor can be preserved with some sulfites, while in red wines, the tannin is an additional preservative that supplements any SO_2_ to also scavenge oxidation and prevent loss of fruity aromas.

The second oxidation product, aldehydes, is dominated by acetaldehyde, since its precursor, ethanol, vastly outnumbers other target alcohols. An aroma of oxidation is present when these aldehyde products accumulate in the absence of SO_2_ [11]. Of the various antioxidants mentioned above, only SO_2_ can really reduce their formation or hide their presence. These are formed by hydrogen peroxide, the other product of the first step of oxidation, and its reaction with iron to form hydroxyl radical [12]. This radical will oxidize pretty much anything present, but as noted above, the preponderance of ethanol means that most of the oxidation will yield acetaldehyde. SO_2_ is able to mitigate formation because it will react quickly with hydrogen peroxide [13], avoiding the radical formation. Of the other antioxidants, glutathione and ascorbate will react with hydrogen peroxide [14], though usually ascorbate is not present at significant levels unless added. However, then, even when some acetaldehyde is formed, the SO_2_ will bind it to make the sulfonate, rendering it and related aldehydes non-volatile. Glutathione and phloroglucinol will also react with acetaldehyde, but only partially, reducing, but not eliminating oxidation aromas [15].

## 3. Oxidation Tests

The basis of the FRAP assay is the conversion of iron (III) to iron (II) a one electron reduction step. The iron (II) is then visualized by complexation with ferrozine, and levels down to 2 µM ascorbic acid equivalent (350 µg/L) can be observed [5]. In wine, the primary reducing species is the catechol functional group, which is converted to the quinone form via a semi-quinone radical. The catechol functional group is abundant on condensed tannin found in red wine, as well in the hydroxycinnamates found in both red and white wine. On a mass or molar basis, such phenolics are by far the most abundant substance in wine that can reduce iron (III). However, other reducing species could also participate in this reduction step, and ascorbic acid is very effective at reducing iron, while the thiol glutathione can reduce iron, but is much weaker than catechols [16]. Sulfite is potent in the FRAP assay [8]. Mono-hydroxy phenols are also fairly ineffective at this reduction, generally at least two orders of magnitude lower, as the monohydroxy compounds do not easily oxidize to the quinone form, and meta-dihydroxy compounds are also weak for the same reason. [17].

However, aside from a direct reaction with iron (III), some antioxidants are good nucleophiles that can react with the immediate oxidation product, the quinones, very quickly yielding a catechol, and reversing the oxidation process. After this reversal, the newly formed catechol can react again, so even nucleophiles that do not react with iron (III) can enhance its reduction almost stoichiometrically. These include the thiols, sulfite and the phloroglucinol functional group found on many flavonoids [18], as well as amino acids [19]. The effectiveness of these various compounds in enhancing the response in an FRAP test will depend on the rate of their reaction with quinones. In other words, will they react in the time frame of the assay? In some cases, this is not well known.

Therefore, these substances should be able to enhance the response of antioxidant tests, even if they do not have a direct response. For instance while glutathione has a weak response in the FRAP assay (0.03), it greatly enhances the response of caffeic acid (1.76). Half doses of each of these two substances combined would be expected to give a response of 0.9 if their combination was additive, but that combination actually gave a response of 2.21, showing how effective glutathione is at reducing quinone back to the catechol [20]. Other have observed a similarly enhanced synergy between a catechol-type compound and glutathione [21].

**Figure 5:**
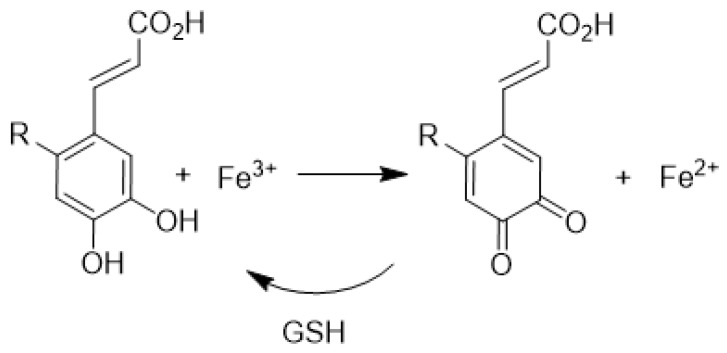
Production of iron (II), the colorimetric product of the FRAP assay, by caffeic acid, and the recycling of the quinone product back to the reactant by glutathione (GSH). This recycling will result in producing more iron (II) per unit of caffeic acid. R=H or GS (glutathionyl).

A similar effect can be observed with different antioxidant tests, but the magnitude of the synergy will vary depending on the capacity of the additive to react directly with the colorimetric reaction [21]. In the Sun example, glutathione has fairly good capacity to quench radicals, so in the DPPH assay, it has a nearly additive effect with caffeic acid. Additionally, ascorbic acid is very effective at reducing iron (III), and so its effect is very nearly additive when combined with other good substrates in the FRAP assay, Figure 5.

In attempting to measure the capacity of a wine to “absorb” oxygen, it is important to distinguish between phenolic substances which are the substrates of oxidation and lead to reactive oxidation intermediates, such as quinones, and those substances which can effectively reverse the oxidation process by quenching those quinones before they can react with flavor molecules. Sulfur dioxide is excellent at this task, as is ascorbate and glutathione. A recent report showed that using LC-MS, it was possible to quantify the amount of substances that would react with quinones by adding a model quinone and looking for all the representative products, the oxidation metabolome [19]. While this provided excellent insight into the inventory of antioxidants, this complex measurement is not practical for production analysis.

## 4. Comparison among Results from FRAP, DPPH, HPLC and FC Assay

Ten high quality, single variety red wine samples vintage 1998 made in Greece were tested after three-years aging. The results in Table 1 of wine made from various grape varieties were significantly different regardless of the identical aging period. It was a good sampling because the range of results on the chemical assays was nearly a factor of two to a factor of 4 for the anthocyanin amounts, and that provided a fairly good range for comparisons to the antioxidant tests seen in Table 2.

In Table 2, the results from FRAP and DPPH showed relatively low correlation with the results from the FC assay (Total phenolics) and total anthocyanins concentration. The results from FRAP and DPPH assay showed relatively high correlation with total flavanols. This suggests that the flavanols in wine are strong contributors to the antioxidant ability of wine.

This correlation is not surprising since the flavanols all contain both a catechol (or galloyl) functional group that can reduce iron (III), or donate a hydrogen to the DPPH radical. In addition, the flavanols contain a phloroglucinol group on the A-ring, which can act as a nucleophile and reduce any oxidized catechol quinone back to the dihydro form by addition, enhancing the antioxidant effect. The correlation with phenolics would be expected to be smaller because some phenolics lack a catechol group, such as coumaric acid or isorhamnetin, and thus cannot reduce iron (III) and the anthocyanins are dominated by malvidin, which lacks a catechol group. Wine also contains other antioxidant substances, such as glutathione, not measured here, but if present, can add noise to a correlation between phenolic assays and antioxidant assays by enhancing the result.

In another study from the same research team, 25 aged red wine (vintage 1998, aged for 7 years) [23] were tested by F-C assay, FRAP assay and DPPH assay as shown in Table 3. The total phenol levels ranged from 1200 to >3700, a range of over three, while the total flavanol levels went from 214 to 922, a more substantial range than the prior study.

As shown in Table 4, the correlation coefficient between total flavanols and FRAP value (r^2^ = 0.6850) was again higher than that of between total phenols and the FRAP value (r^2^ = 0.3884), although the TF-FRAP correlation was weaker than in the earlier study. However, these results still indicate that the flavanols play a major role in the chemical antioxidant capacity of wine, a key factor in the aging of red wine, where the bulk of the phenolic compounds are condensed tannin, made of flavanol units.

In a study of the response of white wines to antioxidant tests, the sulfur dioxide in four white wines were largely removed by adding an aqueous hydrogen peroxide solution [8]. The treated samples were compared with the original wines as shown in Table 5. The treated samples with no free SO_2_ showed much lower FRAP value than original samples. However, DPPH assay and FC assay showed relatively smaller differences between the SO_2_-removed sample and original samples. This suggested that the DPPH value and F-C test were less affected by SO_2_ in samples. After SO_2_ removal, the measurements of DPPH, FRAP, F-C total phenol assay ranked the wine samples similarly. The Sauvignon blanc from South Africa and France showed the highest results, then the Soave from Italy and the Pinot grigio showed the lowest value.

### 4.1. The Age of Wine and Antioxidant Activity

A study of California wines of different ages compared age with various phenolic and antioxidant parameters [25], see Table 6. The wine samples were red wines of 1–28 years old. With increasing wine age, the concentration of monomeric phenolics and LMWP decreased, while the concentration of HMWP increased. However, there was no significant correlation between the wine age and total phenols or antioxidant capacity. The values varied unpredictably with wine age. This study did not look at chemically distinct categories of phenolic compounds, so the phenolic factors that might be affecting these differences could not be assessed.

**Figure 6:**
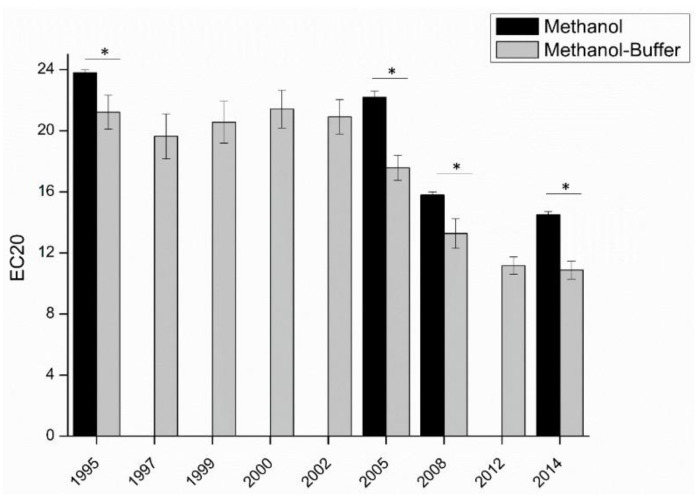
Comparison of the antioxidant capacity of the same appellation from nine vintages from the same winery. Measurements in methanol (black), and in methanol-buffer (grey). Student tests show comparison between methanol and methanol-buffer methods, “*” indicate significant difference (*p* < 0.05). [26].

Another study looking at antioxidant capacity versus vintage age had a different result [26], see Figure 6. The wine samples are white wine of nine vintages from the same vineyard. With increasing wine age, the antioxidant capacity of wine consistently decreased until the wine was about 10 years old. The difference in the results of these two studies is likely due to the fact that the second project had good control over many factors which might influence the wines’ composition because all the grapes were from the same vineyard.

### 4.2. Antioxidant Analysis vs. Sensory Tests

In a diagnostic experiment, wine samples were measured for antioxidant content by a titration, and that result was compared to oxidized character in the wine after forced aging [27]. In Table 7, Group I was the forced aged wine and in Group II were wine of 1–20 years old. The coefficients of correlation between the sensorial (ID) and ROX results from potentiometric titration assay were high for both forced aging wine samples and the age of the group II wine samples. It would appear that the redox titration was a good predictor of resistance to oxidation, albeit under forcing conditions. It would be important to know if this measurement could predict the appearance of oxidized character of wine under normal aging.

## 5. Conclusions

A wine’s aging ability is limited by its oxidation, but can antioxidant assays predict a wine’s aging potential? The various options, such as FRAP, DPPH, voltammetry, and potentiometric titration respond to phenolic compounds as well as protective antioxidants such as SO_2_. Thus, the results of wine antioxidant ability and chemical analysis of polyphenol compounds are correlated, especially for flavanols. Some potentiometric tests showed a good correlation with resistance to oxidation in accelerated aging.

Future work involves testing simple catechols and non-catechol antioxidants with candidate tests, and then with the mixtures to see which can act to synergize their activity in the test beyond the additive effect, by recycling the oxidized form back to the catechol form. Even better would be a test that does not measure catechols, but all the substances that can react with quinones and reverse the oxidation process. The reason for that focus is that the recycling effect is the key to avoiding oxidation, while the catechols are not a strong factor in preventing wine oxidation, but in fact are the pathway to oxidation. A LC-MS assay has recently been shown to document these factors, but the nature of a routine test that could reveal the same is not obvious. A definitive test will be to compare the various assays against the shelf life of a number of commercial wines.

## Figures and Tables

**Table 1:** Polyphenolic Composition and Antioxidant Properties of the Wines Examined [22].

Wine Code	Total Phenolics	Total Flavanols	Total Anthocyanins	A_AR_	SA_HFR_	P_R_
1	2117 ± 117	608.1 ± 3.5	101.8 ± 16.5	1.46 ± 0.07	54.7 ± 0.4	11.03 ± 0.15
2	2276 ± 323	606.5 ± 2.7	81.1 ± 2.5	1.48 ± 0.06	58.0 ± 1.1	10.27 ± 0.21
3	3098 ± 112	473.0 ± 6.6	212.6 ± 2.0	1.15 ± 0.07	47.8 ± 1.3	8.64 ± 0.05
4	3450 ± 171	568.5 ± 1.2	248.4 ± 2.1	1.32 ± 0.07	53.0 ± 0.9	9.06 ± 0.07
5	2898 ± 186	596.7 ± 0.7	363.1 ± 2.4	1.42 ± 0.08	60.0 ± 1.9	10.59 ± 0.05
6	1757 ± 145	347.0 ± 10.9	136.7 ± 5.0	1.10 ± 0.02	43.7 ± 1.5	5.86 ± 0.07
7	1217 ± 292	339.1 ± 6.1	109.7 ± 20.1	0.89 ± 0.06	42.2 ± 0.9	5.36 ± 0.04
8	1328 ± 235	424.4 ± 7.4	271.2 ± 18.5	0.88 ± 0.06	46.8 ± 0.6	5.49 ± 0.09
9	3772 ± 284	664.8 ± 9.5	360.1 ± 9.9	1.53 ± 0.07	61.4 ± 0.5	10.80 ± 0.18
10	3287 ± 250	643.6 ± 6.0	121.7 ± 3.8	1.39 ± 0.04	57.0 ± 1.0	8.35 ± 0.14
ave	2390	527	201	1.26	52.5	8.55

Total phenolics (mg/L gallic acid) by F-C; A_AR_ (mM Trolox) by DPPH assay; P_R_ (mM ascorbic acid) by FRAP assay, total flavanols (mg L-1 catechin) by DMACA method.

**Table 2:** Correlations between DPPH, FRAP assay and total phenolics (TP), total flavanols (TF) and total anthocyanins (TA) concentration. [22].

Values Correlated	Corresponding Correlation Coefficients (r^2^)
DPPH-TP	0.523
FRAP-TP	0.465
DPPH-TF	0.842
FRAP-TF	0.786
DPPH-TA	0.018
FRAP-TA	0.060

**Table 3:** Polyphenolic composition and antioxidant parameters of the wines tested (*n* = 3) [23].

Wine	TP	TF	A_AR_	P_R_
Archanes	3061 ± 211	665.3 ± 11.8	0.98 ± 0.04	8.13 ± 0.12
Archanes	1543 ± 218	302.6 ± 9.5	0.79 ± 0.02	5.80 ± 0.09
Goumenissa	2613 ± 150	546.8 ± 4.4	1.13 ± 0.07	9.04 ± 0.10
Goumenissa	2165 ± 189	214.2 ± 6.1	1.08 ± 0.09	6.49 ± 0.06
Naoussa	2117 ± 117	608.1 ± 3.5	1.46 ± 0.07	11.03 ± 0.15
Naoussa	2276 ± 323	606.5 ± 2.7	1.48 ± 0.06	10.27 ± 0.21
Nemea	3098 ± 112	473.0 ± 6.6	1.15 ± 0.07	8.64 ± 0.05
Nemea	3450 ± 171	568.5 ± 1.2	1.32 ± 0.07	9.06 ± 0.07
Nemea	2898 ± 186	596.7 ± 0.7	1.42 ± 0.08	10.59 ± 0.05
Paros	3606 ± 51	922.3 ± 3.2	1.42 ± 0.06	11.54 ± 0.24
Kritikos Topikos	1939 ± 96	291.1 ± 10.4	0.77 ± 0.00	5.29 ± 0.04
Kritikos Topikos	1658 ± 199	309.7 ± 3.1	0.85 ± 0.01	6.51 ± 0.05
Kritikos Topikos	1709 ± 34	444.7 ± 11.2	0.97 ± 0.02	8.14 ± 0.10
Macedonikos Topikos	1217 ± 292	339.1 ± 6.1	0.73 ± 0.02	5.36 ± 0.04
Peloponissiakos Topikos	2091 ± 57	442.9 ± 3.8	0.87 ± 0.01	7.11 ± 0.07
Peloponissiakos Topikos	1780 ± 181	346.3 ± 11.2	1.04 ± 0.03	8.18 ± 0.22
Topikos Dramas	2439 ± 278	650.4 ± 19.6	1.14 ± 0.05	8.57 ± 0.11
Topikos Chalkidikis	1328 ± 235	424.4 ± 7.4	0.88 ± 0.01	5.49 ± 0.09
Topikos Florinas 19	2187 ± 231	546.8 ± 7.3	1.08 ± 0.02	7.83 ± 0.06
Topikos Imathias	3772 ± 284	664.8 ± 9.5	1.37 ± 0.05	10.80 ± 0.18
Topikos Letrinon	2354 ± 223	352.2 ± 2.4	0.91 ± 0.05	6.63 ± 0.01
Topikos Op. Lokridos	2243 ± 245	435.9 ± 3.0	1.03 ± 0.02	7.39 ± 0.02
Topikos Plagionn Egialias	3287 ± 351	643.6 ± 6.0	1.22 ± 0.01	8.35 ± 0.14
Topikos Stereas Elladas	1943 ± 274	380.2 ± 10.8	0.94 ± 0.02	6.23 ± 0.01
Topikos Tegeas	1995 ± 256	383.0 ± 10.1	0.99 ± 0.02	7.12 ± 0.02
Average	2351	526.8	1.05	8.02

TP: Total phenolics (mg/L gallic acid) by F-C; TF: Total flavanols (mg/L catechin); A_AR_ (mM Trolox) by DPPH assay; P_R_ (mM ascorbic acid) by FRAP assay.

**Table 4:** Statistical parameters as calculated from correlations established using regression analysis at a 99.9% (*p* = 0.001) [23].

Values Correlated	Corresponding Correlation Coefficients (r^2^)
DPPH-TP	0.4420
FRAP-TP	0.3884
DPPH-TF	0.5508
FRAP-TF	0.6850
DPPH-TA	0.1168
FRAP-TA	0.1006

TP (Total phenolics) (mg/L gallic acid) by F-C; AAR (mM Trolox) by DPPH assay; PR (mM ascorbic acid) by FRAP assay, TF (total flavanols).

**Table 5:** Comparison of results obtained with original white wines and after SO_2_ was largely removed [8].

Free and Bound SO_2_ (mg/L)	FRAP		DPPH		F-C	
Sauvignon blanc						
SB-1 Free 33.6 Bound 103.2	225.2 ± 0.3		96.4 ± 1.5		303 ± 9	
SB-2 Free nil Bound 35.5		85.0 ± 1.0		73.2 ± 0.6		225 ± 14
SB-3 Free nil Bound 4.8		84.1 ± 0.4		74.6 ± 1.2		221 ± 2
Sauvignon blanc						
SB-4 Free 14.4 Bound 84.8	174.5 ± 0.3		81.5 ± 3.0		264 ± 4	
SB-5 Free nil Bound 2.4		87.0 ± 0.7		75.0 ± 1.4		225 ± 2
Soave						
S-1 Free 11.2 Bound 84.8	161.7 ± 0.9		79.4 ± 0.6		244 ± 2	
S-2 Free nil Bound 3.2		79.0 ± 1.2		62.3 ± 0.9		202 ± 4
Pinot Grigio						
PG-1 Free 22.4 Bound 85.6	182.6 ± 6		63.0 ± 1.1		247 ± 9	
PG-2 Free nil Bound 3.2		53.2 ± 0.1		54.4 ± 0.3		176 ± 2
Mean	186	78	80	68	264	210

FRAP and DPPH (mg/L Caffeic acid equivalent), F-C (mg/L gallic acid equivalent). Caffeic acid equivalent = (E320*1.4) × 11.1 (mg/L) [24].

**Table 6:** Levels of wine phenolic monomers, low molecular-weight polymers (LMWP) and high molecular-weight polymers (HMWP) as determined by using HLPC and parameters characterizing AOA given by Folin–Ciocalteau total phenols, CV response (Q500) and overall AOA (N) See [25] for details.

Grape and Vintage	Monomers (% Area)	LMWP (% Area)	HMWP (% Area)	FC (mg/L GAE)	Q_500_ (mg/L CE)	N (mM)
Cabernet 1999	20.3	13	66.7	1193	1655	12.8
Cabernet 1999	17.3	12.7	70	2361	3157	27.9
Cabernet 1998	24	13.6	62.4	1162	1366	12
Cabernet 1984	16	8.6	75.4	1897	1493	20.6
Cabernet 1977	9.8	8.2	82	2379	2099	27.9
Cabernet 1973	17.5	9.9	72.6	1002	941	11.6
Zinfandel 1999	20.3	15.2	64.5	2152	2372	24.4
Zinfandel 1999	24.2	16.7	59.1	2457	3336	24.7
Zinfandel 1994	29.4	19.1	51.5	689	752	7.9
Zinfandel 1989	18.6	10.6	70.8	1854	2011	19.8
Pinot Noir 2000	35.2	18.2	46.6	1711	2504	17.8
Pinot Noir 1999	28.5	18	53.5	1693	1890	20.1
Pinot Noir 1998	27	17	56	1563	1180	17.8
Pinot Noir 1992	27.7	16.7	55.6	930	1573	15.6
Pinot Noir 1984	20.5	10.1	69.4	1655	1941	20.4
Merlot 1999	19.9	16.4	63.7	2410	4819	29.3
Merlot 1999	19.3	14.7	66	2616	3949	28.2
Barbera 2001	35.1	19	45.9	1597	1335	17.1
Barbera 1999	34.6	21	44.4	1155	1672	15.6
Grenach 2001	27.3	17.3	55.4	996	1738	18.6
Carmine 1986	17.8	10.7	71.5	980	1165	12

**Table 7:** Coefficients of correlation between sensorial descriptors and dROX Values from Group I and Group II [27].

Corr Coeff (r) with ROX	Wine Group I Forced Aging Experiment (*n* = 13)	Wine Group II Commercial Wines (*n* = 24)
ID	0.8869	0.8725
floral	−0.9068	−0.7728
honey-like	0.8815	0.7826
hay	0.9465	0.8252
woody-like	0.9358	0.8411
farm feed	0.8628	0.8286
